# Differential regulation of SOCS signalling and immunoproteasome pathways by JAK inhibitors and TNF inhibitors in rheumatoid arthritis

**DOI:** 10.1186/s13075-026-03895-7

**Published:** 2026-09-15

**Authors:** Khetam Ghannam, Linh Nguyen My, Christine Behrendt, Monika Brunner-Weinzierl, Anke Lux, Jessica Bertrand, André Wille, Mandy Gläß, Stephan Heinemann-Dammann, Katrin Borucki, Eugen Feist

**Affiliations:** 1 Experimental Rheumatology, Otto-von-Guericke University Magdeburg, Leipziger Str. 44, Magdeburg, 39120 Germany; 2 Experimental Paediatrics and Neonatology, Otto-von-Guericke University Magdeburg, Leipziger Str. 44, Magdeburg, 39120 Germany; 3 Institute for Medical Data Science, Otto-von-Guericke University Magdeburg, Leipziger Str. 44, Magdeburg, 39120 Germany; 4 University Hospital Magdeburg, Orthopedic University Hospital, Leipziger Str. 44, Magdeburg, 39120 Germany; 5 Rheumatology and Clinical Immunology, Helios Clinic Vogelsang-Gommern, Gommern, 39245 Germany; 6 Institute of Clinical Chemistry and Pathobiochemistry, Otto-von-Guericke University Magdeburg, Leipziger Str. 44, Magdeburg, 39120 Germany

**Keywords:** Rheumatoid arthritis, JAK inhibitors, Baricitinib, Tofacitinib, TNF inhibitors, JAK/STAT pathway, SOCS proteins, Immunoproteasome

## Abstract

**Background:**

Janus kinase (JAK) inhibitors and TNF inhibitors (TNFi) are established therapies in rheumatoid arthritis (RA), yet their differential effects on the JAK/STAT/SOCS/proteasome axis remain incompletely understood. This pilot study systematically characterised treatment-specific effects of baricitinib, tofacitinib, and TNFi upon molecular signatures in RA patients.

**Methods:**

Seventy-five patients with RA were enrolled across two prospective cohorts: an initial inception cohort treated with tofacitinib (*n* = 15) and a subsequent confirmatory cohort treated with baricitinib or TNFi (*n* = 60; baricitinib *n* = 29, TNFi *n* = 31), along with 17 and 33 healthy donors (HD)serving as controls for each cohort, respectively. mRNA expression of SOCS1, SOCS2, SOCS3, CIS1, and the immunoproteasome subunit β2i was assessed in peripheral blood mononuclear cells by real-time PCR. SOCS2 protein levels were measured by ELISA, and serum IL-6 levels were evaluated in the baricitinib/TNFi cohort.

**Results:**

Responder rates were comparable across all three treatment groups at both time points. JAK inhibition was associated with a distinct SOCS suppression signature: SOCS2 mRNA declined progressively under both baricitinib and tofacitinib but not under TNFi blockade. SOCS1 and SOCS3 mRNA normalized under baricitinib over time, while SOCS3 elevation persisted throughout the observation period under TNFi. CIS1 was selectively suppressed under baricitinib. Partial discordance between SOCS2 mRNA and protein levels suggested post-transcriptional regulation. Baseline SOCS3 expression was significantly higher in baricitinib non-responders than responders, and early SOCS3 decline at 2 weeks distinguished TNFi responders from non-responders. Proteasomal β2i mRNA was relatively upregulated under tofacitinib and TNFi but not under baricitinib.

**Conclusion:**

JAK inhibition drives a treatment-specific suppression of SOCS family members not observed under TNFi, reflecting differential remodelling of cytokine feedback networks. Baseline and early SOCS3 dynamics emerge as candidate biomarkers of treatment response. These findings may inform future efforts to personalise treatment selection in RA and warrant validation in larger, randomised cohorts.

## Background

Rheumatoid Arthritis (RA) is a chronic systemic autoimmune disease characterised by persistent synovial inflammation, progressive cartilage destruction, and bone erosion, ultimately leading to pain, disability, and reduced quality of life [1, 2]. The pathogenesis of RA is driven by a complex network of pro-inflammatory cytokines, including tumour necrosis factor alpha (TNF-α), interleukin-6 (IL-6), interferons (IFNs), and IL-17, which collectively sustain immune activation and chronic inflammation within the synovium [1, 3]. Many of these cytokines signal through the Janus kinase/signal transducer and activator of transcription (JAK/STAT) pathway, a central regulator of immune responses and inflammatory gene expression [3, 4].

The JAK/STAT pathway is counter-regulated by suppressor of cytokine signalling (SOCS) proteins, a family of intracellular negative feedback regulators induced by cytokine stimulation [5, 6]. SOCS proteins inhibit cytokine signalling by blocking JAK activity and promoting ubiquitin-mediated proteasomal degradation of signalling components [6, 7]. This regulatory loop implicates the proteasome as a critical effector of JAK/STAT pathway inactivation. Under the chronically pro-inflammatory conditions of RA, sustained cytokine exposure drives remodelling of the standard proteasome into the immunoproteasome through replacement of constitutive catalytic subunits (β1, β2, β5) by inducible counterparts (β1i, β2i, β5i), enhancing antigen processing efficiency while simultaneously modulating SOCS-dependent JAK protein degradation [8, 9]. Dysregulation of SOCS expression has been documented in the synovial tissue and peripheral blood of RA patients, implicating impaired feedback control as a contributor to sustained JAK/STAT activation in this disease [10, 11].

JAK inhibitors (JAKi) have emerged as highly effective targeted therapies in RA. Tofacitinib, the first approved agent in this class, broadly inhibits JAK1–3 with some TYK2 activity, simultaneously impairing signalling through multiple cytokine axes [1]. Baricitinib offers more selective JAK1/JAK2 inhibition, with particular potency against IL-6 and interferon receptor signalling [12]. Both agents have demonstrated efficacy comparable to or exceeding adalimumab in randomised controlled trials [12, 13]. TNF-α inhibitors (TNFi), by contrast, act upstream of JAK/STAT through TNFR-mediated signalling, leaving IL-6 and other JAK-dependent cytokine pathways largely intact [14]. Notably, baricitinib has demonstrated pronounced analgesic effects that emerge within days of treatment initiation and appear to exceed those observed with TNF-α inhibitors — an observation that suggests modulation of additional molecular pathways, potentially including neuroinflammatory JAK signalling in dorsal root ganglia and spinal cord, not yet fully elucidated [12, 15].

Despite the established clinical profiles of these agents, their differential effects on the SOCS/proteasome regulatory axis in circulating immune cells remain incompletely characterised. Understanding treatment-specific molecular signatures is of growing importance in the context of precision medicine in RA, where identifying predictive biomarkers may inform future treatment selection [16]. To characterise and compare the molecular effects of these treatment modalities, we conducted a prospective pilot study examining the JAK/STAT/SOCS/proteasome axis in tofacitinib-treated patients, subsequently extending the analysis to cohorts treated with baricitinib and TNFi as a comparator arm.

## Methods

### Patients

#### Tofacitinib cohort

In an initial inception pilot study, 15 patients with RA (73% female and 27% male with mean age 62 years) were investigated over a follow-up period of 24 weeks and compared to 17 healthy matched controls. Blood samples were collected at three time points: Baseline before treatment initiation (BL), and during treatment at weeks 12 (visit 1, V1) and 24 (visit 2, V2). All patients were recruited from the Department of Rheumatology, Helios Clinic Vogelsang-Gommern, Germany. The molecular findings emerging from this cohort prompted the design of a subsequent confirmatory study to validate and extend the observations to additional treatment modalities.

#### Baricitinib and TNFi cohorts

Based on the results of the tofacitinib cohort, a subsequent prospective confirmatory study was conducted, enrolling 60 patients with RA at the Department of Rheumatology, Helios Clinic Vogelsang-Gommern, in cooperation with the Departments of Experimental Rheumatology and Experimental Paediatrics, Otto-von-Guericke University Magdeburg. This second cohort was designed to confirm the tofacitinib findings with baricitinib — a more selective JAK1/2 inhibitor — and to compare JAK inhibitor-specific effects against TNFi as a mechanistically distinct comparator. A control group of 33 healthy donors (HD) was included for biomarker analyses. Of the 60 patients, 29 were treated with baricitinib (72% female and 28% male with mean age 58) and 31 received a TNFi (74% female and 26% male with mean age 61 years). The TNFi-treated group included patients receiving etanercept (*n* = 26) or adalimumab (*n* = 5). Blood samples were collected at three time points: baseline before treatment initiation (BL), and during treatment at weeks 2 (visit 1, V1) and week 12 (visit 2, V2).

All patients were treated according to the approved label and routine clinical practice. Both studies were approved by the Ethics Committee of the Otto-von-Guericke University, Magdeburg, Germany, and written informed consent was obtained from all participants prior to inclusion. The demographic and baseline clinical characteristics of the study cohorts are summarized in Table 1.

**Table 1:** The demographic and baseline clinical characteristics of the study cohorts

Characteristic	TNF inhibitors (*n* = 31)	Baricitinib (*n* = 29)	Tofacitinib (*n* = 15)
Female, n (%)	23 (74.2%)	21 (72.4%)	11 (73.3%)
Male, n (%)	8 (25.8%)	8 (27.6%)	4 (26.7%)
Age, female (years)	62.3 ± 10.9	57.5 ± 11.8	65.3 ± 7.8
Age, male (years)	55.9 ± 14.3	57.8 ± 8.5	54.5 ± 15.3
Prednisolone therapy, n (%)	16 (51.6%)	20 (69.0%)	8 (53.33%)
Methotrexate therapy, n (%)	9 (29.0%)	7 (24.1%)	2 (13.33%)
Opioid use, n (%)	0 (0%)	3 (10.3%)	2 (13.33%)
NSAID use, n (%)	11 (35.5%)	18 (62.1%)	2 (13.33%)
Previous biologic therapy, n (%)	4 (12.9%)	22 (75.9%)	10 (66.67%)
Disease duration (years)	6.49 ± 6.49	10.26 ± 9.95	13.78 ± 10.78
Baseline DAS28-ESR	5.58 ± 1.10	4.98 ± 1.42	5.57 ± 1.11

#### Disease activity assessment

Disease activity was evaluated using the 28-joint Disease Activity Score based on erythrocyte sedimentation rate (DAS28-ESR), in accordance with the European League Against Rheumatism (EULAR) response criteria [17, 18]. Disease activity was categorized as follows: remission (DAS28 < 2.6), low (DAS28 ≤ 3.2), moderate (3.2 < DAS28 ≤ 5.1), and high (DAS28 > 5.1). Treatment response was defined as an improvement in DAS28-ESR of more than 1.2 points from baseline to either visit 1 (V1) or visit 2 (V2) [18].

#### Isolation of peripheral blood mononuclear cells (PBMCs)

Peripheral blood mononuclear cells (PBMCs) from whole blood collected in heparin tubes were separated through density gradient centrifugation using Ficoll separating solution with the density of 1.077 g/ml (Biochrom, Berlin, Germany). The collected cells were washed and half of the cells were used for RNA isolation, the other half was lysed in NP-40 for protein analysis.

For serum preparation, peripheral blood was collected into serum collection tubes and allowed to clot at room temperature for 30 min. Samples were then centrifuged at 2,000 × *g* for 10 min, and the serum was collected, aliquoted, and stored at − 80 °C until analysis. Serum IL-6 concentrations were measured in the baricitinib- and TNFi-treated patient cohorts using the Bio-Plex Pro Human Chemokine IL-6 Set (Bio-Rad, Hercules, CA, USA; catalogue no. 171BK29MR2) according to the manufacturer’s instructions. IL-6 was selected as a representative downstream biomarker of JAK/STAT pathway activation because it signals predominantly through the JAK1/JAK2–STAT3 pathway and plays a central role in the pathogenesis of rheumatoid arthritis. Serum IL-6 levels were therefore assessed to complement the gene expression analyses by providing a measure of systemic inflammatory activity. Serum samples were not available from the tofacitinib cohort; therefore, IL-6 measurements could not be performed in these patients.

#### RNA isolation and reverse transcription into cDNA

PBMCs were lysed in Trizol reagent (Thermo Fisher Scientific, Carlsbad, USA) at a ratio of 500 µl Trizol per 3–7 × 10^5^ PBMCs. The lysate was then centrifuged at 13,000 × g for 10 min at 4 °C. To the supernatant, 100 µl of chloroform (Merk Millipore, Darmstadt, Germany) was added and incubated on ice for 2 min, followed by centrifugation at 10,000 × g for 15 min at 4 °C. The upper aqueous phase containing RNA was carefully separated from the rest of the mixture and the RNA was extracted using the Nucleospin RNA kit (Macherey–Nagel, Düren, Germany) according to the manufacturer’s guidelines. Quality and quantity of RNA were controlled using a NanoDrop 1000 spectrophotometer (Thermo Scientific, Germany). To synthesize first-strand cDNA from total RNA, SuperScript™ III First-Strand Synthesis System for RT-PCR (Thermo Fisher Scientific, Germany) was used according to the instructions of the manufacturer. The cDNA was used as a template for the specific primers in real time PCR (rt-PCR).

#### Gene expression analysis by real-time reverse transcriptase-polymerase chain reaction (real time RT-PCR)

Real time RT-PCR was used for the relative quantification of gene expression across the proteasome system and JAK/STAT signalling pathway.

In the tofacitinib cohort, expression of the following genes was investigated: the three constitutive catalytic proteasome subunits (β1, β2, β5) and their corresponding immunoproteasome counterparts (β1i, β2i, β5i); all four JAK family members (JAK1, JAK2, JAK3, TYK2); STAT family members (STAT1, STAT3, STAT4, STAT5A, STAT5B, STAT6); and the SOCS family members SOCS1, SOCS2, SOCS3, and CIS1.

As no significant differences were observed for most analysed genes between patients and HD or across different time points within patients, subsequent analyses in the baricitinib and TNFi cohorts were focused on the most informative molecular targets identified in the inception tofacitinib cohort. Accordingly, gene expression analysis in these cohorts was restricted to SOCS family members (SOCS1, SOCS2, SOCS3, and CIS1) and the immunoproteasome subunit β2i.

Amplification reactions were performed using SYBR Green PCR Master Mix (Thermo Fisher Scientific, Germany) with 200 nM forward and reverse primers for each gene, together with the corresponding cDNA template. Real-time PCR was carried out in triplicate on a StepOnePlus Real-Time PCR System (Thermo Fisher Scientific, Germany).

#### Normalization and housekeeping genes

Relative gene expression was normalised using three candidate housekeeping genes: beta-actin (ACTB), glyceraldehyde-3-phosphate dehydrogenase (GAPDH), and hypoxanthine–guanine phosphoribosyltransferase 1 (HPRT1). Forward and reverse primer sequences were designed as previously described [10, 19, 20]. The sequences of all primers used in this study are provided in Table 2.

**Table 2:** Primer sequences used in real-time PCR

Nr	Gene	Forward primer sequence (5'- 3')	Reverse primer sequence (5'- 3')
1	**SOCS1**	ctgggatgccgtgttatttt	taggaggtgcgagttcaggt
2	**SOCS2**	cagggaatggcagagacact	tggcagagagagaagggatg
3	**SOCS3**	gccacctactgaaccctcct	acggtcttccgacagagatg
4	**CIS1**	agcccagacagagagtgagc	tgacagcgtgaacaggtagc
5	**STAT1**	tcacattcacatgggtggag	caaaggcatggtctttgtca
6	**STAT3**	tcacatgccactttggtgtt	gcaatctccattggcttctc
7	**STAT4**	ggcaattggagaaactagagg	agggtgggttggcatacat
8	**STAT5A**	gccagatgcaggtgctgta	gggattgtccaagtcaatgg
9	**STAT5B**	gcgttatatggccagcattt	ctggtgctctgccttcttct
10	**STAT6**	ggaagggcactgagtctgtc	ggctttggcattgttgtctt
11	**JAK1**	catggtggaagagtttgtgga	cagctgtttggcaactttgaatt
12	**JAK2**	ccgccgggtttcagaag	gaagaggtggatgttccctcc
13	**JAK3**	agtgggactttcctctcgc	ctcttcacttggaggtgccat
14	**TYK2**	cccatggcttggaagatggt	actcagcttgatgaaggggc
15	**Beta-1**	caagctgacacctattcacgac	cggtatcggtaacacatctcct
16	**Beta-1i**	caacgtgaaggaggtcaggta	agagcaatagcgtctgtggtg
17	**Beta-2**	atcgctggggtggtctataag	aagaaatgagctgggttgtcat
18	**Beta-2i**	aatgtggacgcatgtgtgat	catagcctgcacagtttcctc
19	**Beta-5**	ggcaatgtcgaatctatgagc	gttcccttcactgtccacgta
20	**Beta-5i**	cacgggtagtgggaacactta	actttcacccaaccatcttcc
21	**Beta-actin**	ctggacttcgagcaagagatg	tgaaggtagtttcgtggatgc
22	**GAPDH**	atgttcgtcatgggtgtgaa	acagtcttctgggtggcagt
23	**HPRT1**	cgtgattagtgatgatgaaccag	cacagagggctacaatgtgatg

The expression stability of the candidate housekeeping genes was evaluated using the geNorm [21] and NormFinder [22] algorithms according to the developers’ recommendations. For each housekeeping gene, the mean Ct value of each sample was calculated and converted into relative quantities (RQ) using the comparative Ct method. For each gene, the lowest Ct value across all samples was used as the calibrator according to the following equation:

$$\begin{aligned}&\mathrm{RQ}=\mathrm E^{\mathrm{Ct}\;\min\;\mathrm{housekeeping}\;\mathrm{gene}}/\\&\mathrm E^{\mathrm{Ct}\;\mathrm{housekeeping}\;\mathrm{gene}\;\mathrm{in}\;\mathrm{sample}}\end{aligned}$$

where E represents the amplification efficiency of the respective primer pair, Ct min is the lowest Ct value observed for each housekeeping gene across all sample, and Ct sample is the Ct value of the respective sample. The resulting RQ values were used as input for both geNorm and NormFinder analyses.

Target gene expression was subsequently normalized to the selected housekeeping gene and calculated according to the Pfaffi method R = E^Ct housekeeping gene^/E^Ct target gene^ [23] where E represents the amplification efficiency of the corresponding primer pair.

#### Identification of the most stable housekeeping gene

The geNorm algorithm ranks candidate reference genes according to their average pairwise expression stability (M value), with lower M values indicating greater stability. NormFinder uses a model-based approach to estimate both intra- and intergroup variation, generating a stability value in which lower values also indicate more stable expression.

Both algorithms consistently identified ACTB as the most stable housekeeping gene. geNorm ranked ACTB highest (M = 1.31), followed by GAPDH (M = 1.58) and HPRT1 (M = 2.04). Similarly, NormFinder identified ACTB as the most stable reference gene (stability value = 0.099), followed by GAPDH (0.178) and HPRT1 (0.357). Although the combination of ACTB and GAPDH showed slightly improved stability, ACTB alone was selected as the reference gene for relative quantification because it exhibited the highest stability across both algorithms.

The ranking order of the three candidate housekeeping genes according to geNorm and NormFinder is summarized in Table 3.

**Table 3:** Ranking of the candidate housekeeping genes according to their expression stability values calculated by geNorm, NormFinder algorithms

Ranking order	Gene	GeNorm	NormFinder
	Symbol	Expression stability (M)	Stability value
1	ACTB	1.31	0.099
2	GAPDH	1.58	0.178
3	HPRT1	2.04	0.357

#### Measurement of SOCS2 by Enzyme-linked Immunosorbent Assay (ELISA)

PBMCs were lysed in NP-40 lysis buffer, and total protein concentrations were determined using a NanoDrop 1000 spectrophotometer (Thermo Fisher Scientific, Germany). SOCS2 concentrations in PBMC lysates from patients and HD were measured using a human SOCS2 ELISA kit (Antibodies.com, Cambridge, UK) according to the manufacturer’s instructions. Equal amounts of total protein (50 µg per sample) were used for each ELISA measurement to normalize SOCS2 protein levels. SOCS2 protein expression was assessed in both patient cohorts.

### Statistical analysis

The non-parametric Wilcoxon matched-pairs signed-rank test was used to analyse the effects of baricitinib, tofacitinib, and TNFi on the expression of the analysed genes. To compare responders and non-responders, changes in gene expression between time points (T1–T2 and T1–T3) were calculated for each gene. These differences were subsequently compared between groups using the Mann–Whitney U test.

Additionally, differences in gene expression between HD and patients at various time points were analysed using the Mann–Whitney U test. A contingency Fisher’s exact test was applied to assess differences in response rates among the three treatment groups.

Correlation analyses were performed using Spearman’s rank correlation coefficient (Spearman’s ρ) to assess associations between changes in gene expression (Δ expression) and changes in clinical parameters (ΔDAS28-ESR and ΔHAQ). Analyses were performed separately for the first (V1) and second (V2) follow-up visits and stratified by treatment response (responders and non-responders) within the baricitinib and TNFi cohorts. The tofacitinib cohort was excluded from these analyses because of the limited number of responders and non-responders.

*P*-values < 0.05 were considered statistically significant. Non-parametric tests were used to determine and verify significance levels. Four levels of significance were defined: *p* < 0.05 (*)*, p* < 0.01 (**)***,**** p* < 0.001 (***), and *p* < 0.0001 (****).

All statistical analyses were performed using SPSS version 29.0.2.0 (IBM Corp.) and GraphPad Prism version 10.0.0.

## Results

### Response rate according to DAS28-ESR

A total of 75 patients were enrolled: 15 in the Tofacitinib group, 29 in the Baricitinib group, and 31 in the TNFi group. At Visit 1, 65 patients were assessed — 12 (80%), 26 (90%), and 27 (87%) from each group respectively; 2 TNFi patients with missing data were excluded from response analysis at this timepoint. At Visit 2, 57 patients completed assessment — 7 (47%), 24 (83%), and 26 (84%) from the Tofacitinib, Baricitinib, and TNFi groups, respectively.

Fisher’s exact tests of independence revealed no statistically significant difference in responder rates among Tofacitinib, Baricitinib, and TNFi at either Visit 1 (50% vs. 46% vs. 44%; *p* > 0.99) or Visit 2 (57% vs. 63% vs. 62%; *p* > 0.99), indicating comparable response proportions across all three treatment groups at both assessment time points.

Longitudinal analysis demonstrated significant reductions in DAS28-ESR in all treatment groups. Tofacitinib treatment significantly reduced DAS28-ESR at Visit 1 (*p* = 0.025) and Visit 2 (*p* = 0.016), although no additional improvement was observed between visits. Baricitinib treatment resulted in significant improvement from baseline to Visit 1 (*p* = 0.024) and Visit 2 (*p* < 0.001), with further improvement between Visit 1 and Visit 2 (*p* = 0.019). TNFi treatment also significantly reduced DAS28-ESR at both follow-up visits (*p* < 0.001), demonstrating sustained clinical efficacy. Detailed clinical response data are summarized in Table 4.

**Table 4:** Comparison of Tofacitinib, Baricitinib, and TNFi— mRNA, serum and protein expression at Baseline, visit 1 (V1) and visit 2 (V2).; — = not significant, HD: Healthy donors, nm: not measured

Parameter	Tofacitinib	Baricitinib	TNF inhibitors
Mann–Whitney Test — Patients vs. Healthy Donors (HD), mRNA and Protein			
SOCS1 mRNA			
SOCS1 Baseline × HD	*P* = 0.0067 ** **↑**in patients	*P* = 0.0038 ** **↑** in patients	—
SOCS1 V1 × HD	—	—	—
SOCS1 V2 × HD	—	—	*P* = 0.032 * **↑** in patients
SOCS2 mRNA			
SOCS2 Baseline × HD	—	—	—
SOCS2 V1 × HD	*P* = 0.0270 * **↓** in patients	*P* = 0.0068 ** **↓** in patients	—
SOCS2 V2 × HD	—	*P* < 0.0001 **** **↓** in patients	—
SOCS3 mRNA			
SOCS3 Baseline × HD	*P* = 0.052 **↑** in patients (n.s.)	*P* < 0.0001 **** **↑** in patients	*P* < 0.0001 **** **↑** in patients
SOCS3 V1 × HD	—	—	*P* = 0.0014 ** **↑** in patients
SOCS3 V2 × HD	—	—	*P* < 0.0001 **** **↑** in patients
CIS1 mRNA			
CIS1 Baseline × HD	—	—	—
CIS1 V1 × HD	—	*P* = 0.0013 ** ↓ in patients	—
CIS1 V2 × HD	—	*P* = 0.0012 ** ↓ in patients	—
Beta2i mRNA			
Beta2i Baseline × HD	—	—	*P* = 0.0053 ** **↓** in patients
Beta2i V1 × HD	*P* = 0.0355 * **↑** in patients	—	*P* = 0.0410 * ↓ in patients
Beta2i V2 × HD	—	—	—
SOCS2 Protein			
SOCS2 protein Baseline × HD	*P* = 0.0389 * **↓** in patients	—	*P* = 0.0075 ** **↓** in patients
SOCS2 protein V1 × HD	—	—	*P* = 0.0153 * **↓** in patients
SOCS2 protein V2 × HD	—	*P* = 0.0312 **↓** in patients	*P* = 0.0476 * **↓** in patients
Wilcoxon Matched-Pairs Signed Rank Test — Longitudinal Changes within Patients, mRNA, Serum and Protein			
DAS28-ESR			
Baseline → V1	*P* = 0.025 * **↓**	*P* = 0.0245 * **↓**	*p* < 0.001 *** **↓**
Baseline → V2	*P* = 0.016 * **↓**	*p* < 0.001 *** **↓**	*p* < 0.001 *** **↓**
V1 → V2	—	*P* = 0.019 * **↓**	—
IL-6 (serum)			
Baseline → V1	nm	*P* = 0.0315 * **↓**	*P* = 0.0522 **↓** (n.s.)
Baseline → V2	nm	*P* = 0.0053 ** **↓**	*P* = 0.0587 **↓** (n.s.)
V1 → V2	nm	—	—
SOCS1 mRNA			
Baseline → V1	—	—	—
Baseline → V2	—	*P* = 0.0327 * **↓**	—
V1 → V2	—	—	—
SOCS2 mRNA			
Baseline → V1	*P* = 0.0059 ** **↓**	*P* < 0.0001 **** **↓**	—
Baseline → V2	*P* = 0.0625 **↓** (n.s.)	*P* = 0.0001 *** **↓**	—
V1 → V2	—	—	—
SOCS3 mRNA			
Baseline → V1	—	*P* = 0.0075 ** **↓**	—
Baseline → V2	—	—	—
V1 → V2	—	—	—
CIS1 mRNA			
Baseline → V1	—	—	—
Baseline → V2	—	*P* = 0.014 * **↓**	—
V1 → V2	—	—	—
Beta2i mRNA			
Baseline → V1	—	—	—
Baseline → V2	—	—	—
V1 → V2	—	—	—
SOCS2 Protein			
SOCS2 protein Baseline → V1	—	—	—
SOCS2 protein Baseline → V2	—	—	—
SOCS2 protein V1 → V2	—	*P* = 0.0595 (n.s., trend increase in V1)	—

*p < 0.05, **p < 0.01, ***p < 0.001, ****p < 0.0001. ↑ increase, ↓ decrease

### Serum IL-6 reduction reflects treatment-associated inflammatory modulation

Serum IL-6 concentrations were evaluated in the baricitinib and TNFi cohorts, as serum samples were not available from the historical tofacitinib cohort. Baricitinib treatment resulted in a progressive reduction of IL-6 levels, reaching statistical significance at Visit 1 (*p* = 0.0315) and further decreasing at Visit 2 (*p* = 0.005). In contrast, TNFi treatment showed only a non-significant tendency toward reduced IL-6 concentrations at Visit 1 (*p* = 0.0522) and Visit 2 (*p* = 0.0587).

The pronounced reduction of IL-6 under baricitinib treatment is consistent with inhibition of IL-6 receptor-associated JAK1/JAK2 signalling, whereas the weaker effect observed under TNFi treatment may reflect indirect modulation of IL-6 production. IL-6 results are shown in Table 4.

### Treatment-associated modulation of SOCS signalling and immunoproteasome expression

In the inception cohort, mRNA expression analysis demonstrated that, except for the SOCS family members (SOCS1, SOCS2, SOCS3, and CIS1) and the immunoproteasome subunit β2i, none of the investigated genes showed significant differences either between patients and healthy donors or across longitudinal time points within patients. Based on these findings, subsequent analyses in the baricitinib and TNFi cohorts were restricted to SOCS family members and β2i expression.

### SOCS1 mRNA expression is reduced following JAK inhibition

**Fig. 1:**
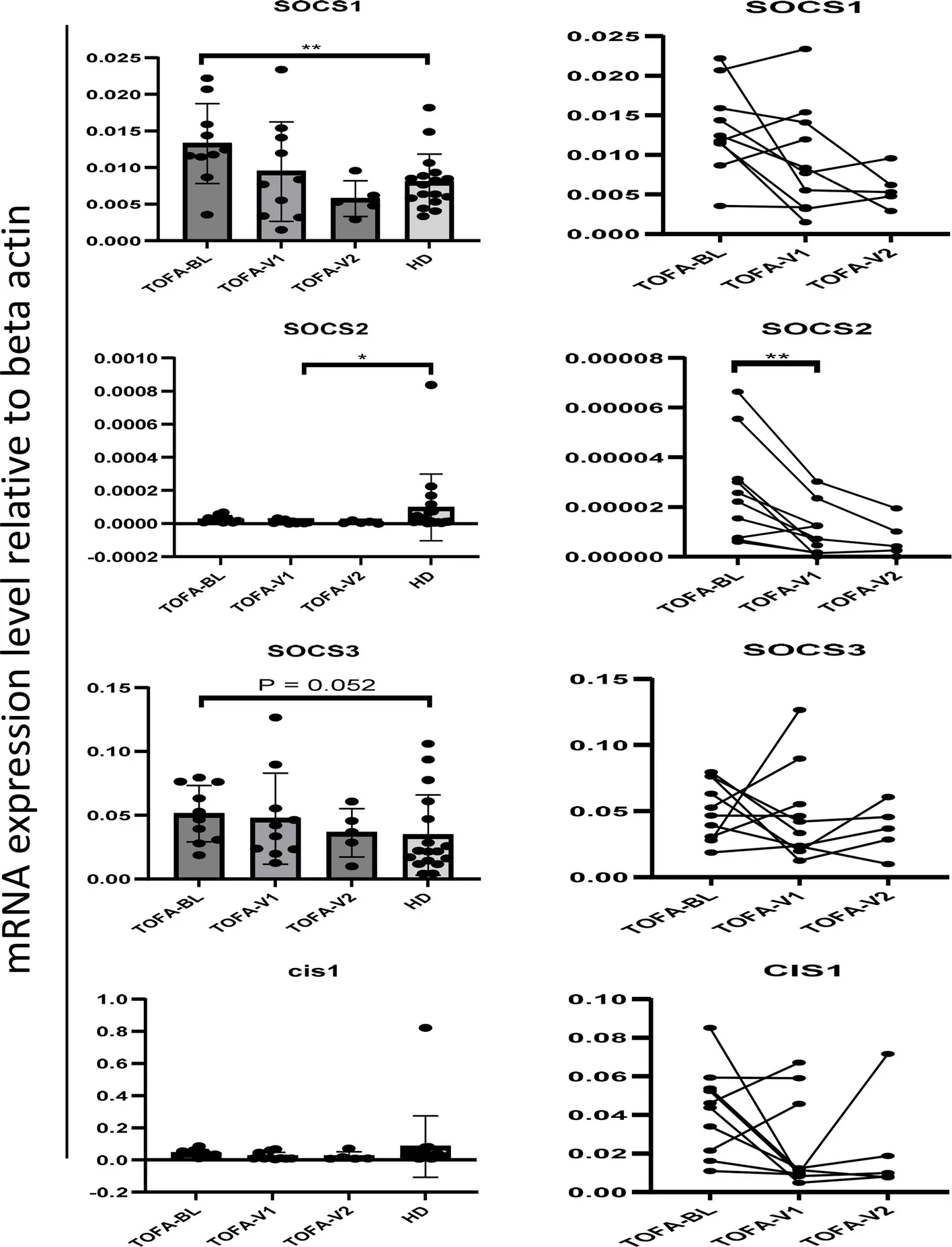
SOCS mRNA Expression Under Tofacitinib Therapy. The Mann–Whitney U test was used to compare SOCS mRNA expression levels between rheumatoid arthritis (RA) patients treated with tofacitinib and healthy donors (HD). The Wilcoxon matched-pairs signed-rank test was applied to assess changes in SOCS expression within patients between baseline (BL), visit 1 (V1; 12 weeks), and visit 2 (V2; 24 weeks). Data are presented as mean ± SD. *P*-values < 0.05 were considered statistically significant. Significance levels are indicated as follows: *p < 0.05, **p < 0.01

**Fig. 2:**
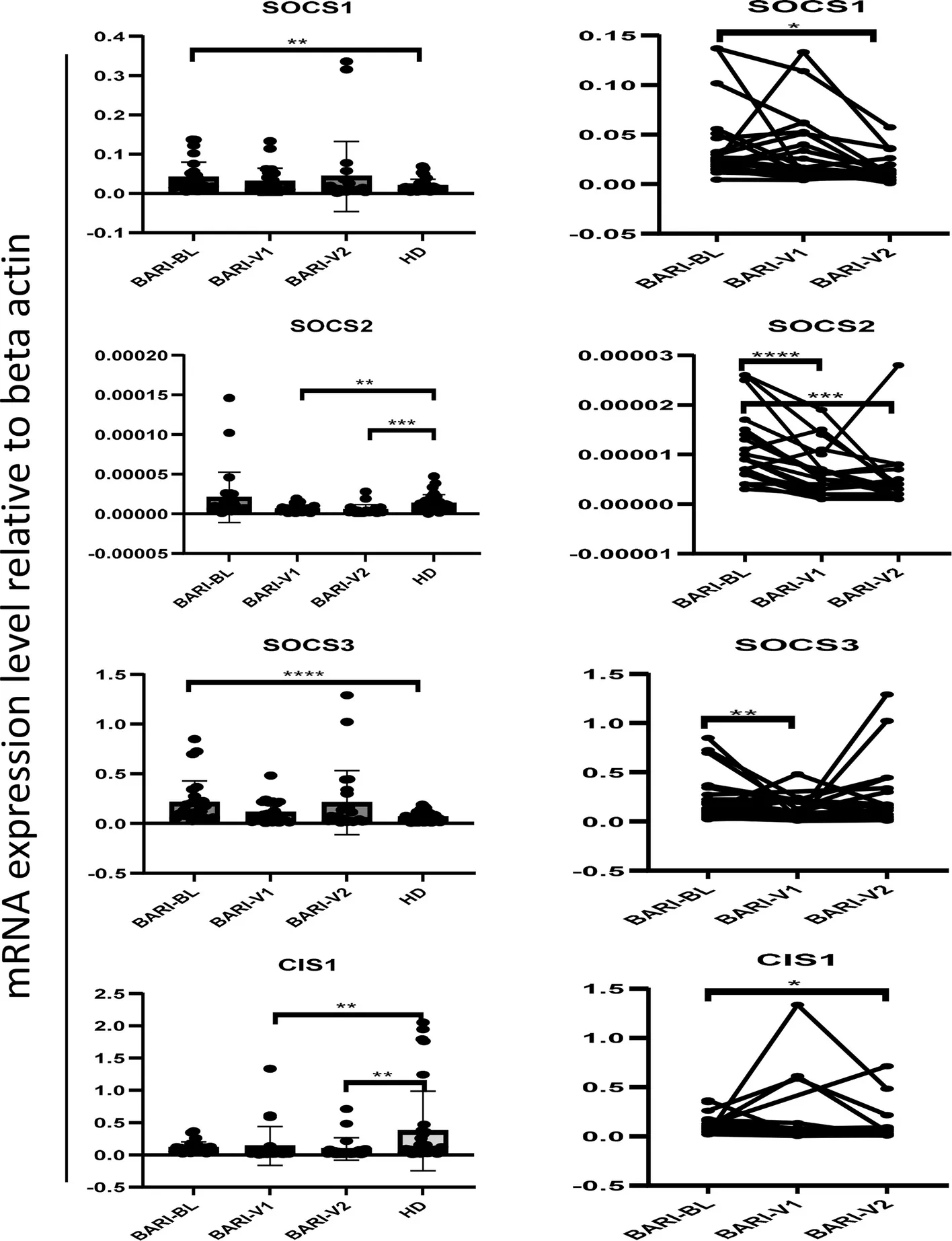
SOCS mRNA Expression Under Baricitinib Therapy. The Mann–Whitney U test was used to compare SOCS mRNA expression levels between rheumatoid arthritis (RA) patients treated with baricitinib and healthy donors (HD). The Wilcoxon matched-pairs signed-rank test was applied to assess changes in SOCS expression within patients between baseline (BL), visit 1 (V1; 2 weeks), and visit 2 (V2; 12 weeks). Data are presented as mean ± SD. *P*-values < 0.05 were considered statistically significant. Significance levels are indicated as follows: *p < 0.05, **p < 0.01, ***p < 0.001, and ****p < 0.0001

**Fig. 3:**
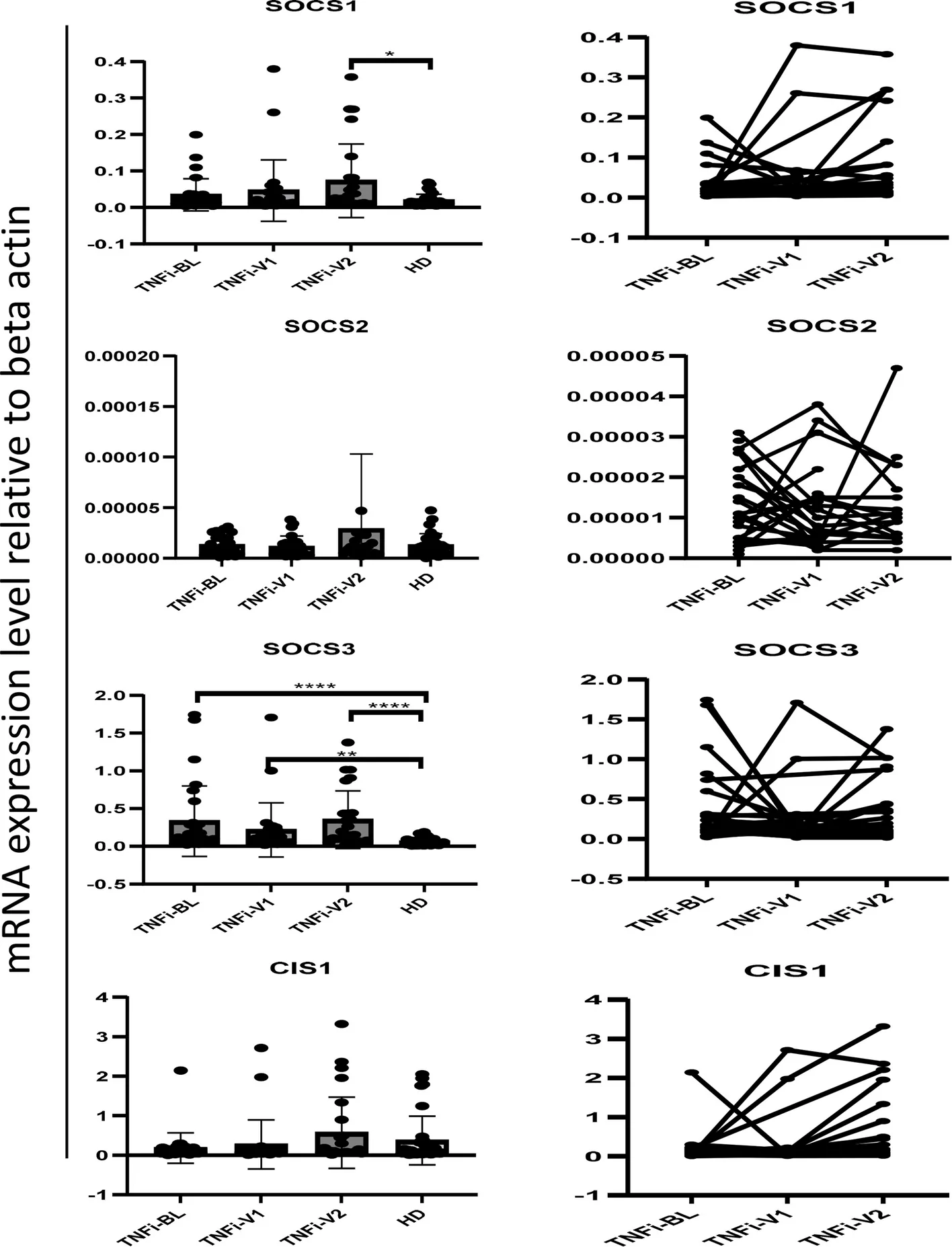
SOCS mRNA Expression Under TNF Inhibitors (TNFI) Therapy. The Mann–Whitney U test was used to compare SOCS mRNA expression levels between rheumatoid arthritis (RA) patients treated with TNFi and healthy donors (HD). The Wilcoxon matched-pairs signed-rank test was applied to assess changes in SOCS expression within patients between baseline (BL), visit 1 (V1; 2 weeks), and visit 2 (V2; 12 weeks). Data are presented as mean ± SD. *P*-values < 0.05 were considered statistically significant. Significance levels are indicated as follows: *p < 0.05, **p < 0.01, and ****p < 0.0001

At baseline, SOCS1 mRNA was significantly elevated in patients compared to HD under both baricitinib (*p* = 0.0038) and tofacitinib (*p* = 0.0067), suggesting pre-treatment immune activation affecting this negative feedback regulator. Notably, within baricitinib-treated patients, SOCS1 mRNA decreased significantly by V2 compared with baseline (*p* = 0.0327), implying that JAK inhibition progressively normalises this pathway (Figs. 1, 2 and 3, Table 4). Under TNFi therapy, SOCS1 was non-significantly elevated at baseline and became significantly elevated at V2 (*p* = 0.032), indicating sustained upregulation compared with HD.

### SOCS2 mRNA represents a prominent JAK inhibitor-associated molecular signature

SOCS2 shows the most consistent treatment-driven changes. Both baricitinib and tofacitinib significantly reduced SOCS2 mRNA compared to HD at V1 (*p* = 0.0068 and 0.027, respectively, with baricitinib maintaining this suppression at V2 (*p* < 0.0001). Within-patient analyses confirm a strong and progressive decline under baricitinib (Baseline to V1: *p* < 0.0001; Baseline to V2: *p* = 0.0001). Tofacitinib shows a similar but attenuated trend (Baseline to V1: *p* = 0.0059; Baseline to V2: *p* = 0.0625, n.s.). TNFi show no significant effect on SOCS2 mRNA (Figs. 1, 2 and 3, Table 4). This suggests that SOCS2 suppression is a JAK inhibitor-specific effect, potentially reflecting reduced cytokine-driven transcription.

### SOCS3 mRNA demonstrates differential regulation according to treatment modality

SOCS3 mRNA is markedly elevated at baseline in both baricitinib and TNFi—treated patients (*p* < 0.0001 for both), with a trend observed in tofacitinib group (*p* = 0.052) compared with HD. Under baricitinib, SOCS3 expression significantly decreased within patients from baseline to V1 (*p* = 0.0075), and expression levels were no longer significantly different from HD at subsequent visits, indicating normalization of SOCS3 expression. In contrast, TNFi -treated patients maintained significantly elevated SOCS3 expression at Visit 1 (*p* = 0.0014) and Visit 2 (*p* < 0.0001), suggesting persistent SOCS3 pathway activation despite clinical improvement (Figs. 1, 2 and 3, Table 4). These findings indicate distinct effects of JAK inhibition and TNFi on SOCS3 regulation and prompted further investigation of SOCS3 as a potential marker associated with treatment response.

### CIS1 and beta2i mRNA expression show treatment-specific modulation

CIS1 mRNA expression was significantly reduced compared to HD specifically under baricitinib treatment at V1 (*p* = 0.0013) and V2 (*p* = 0.0012). Within-patient analyses further confirmed a significant decline under baricitinib (baseline to V2: *p* = 0.014), whereas no significant changes were observed under tofacitinib or TNFi therapy (Figs. 1, 2 and 3, Table 4).

**Fig. 4:**
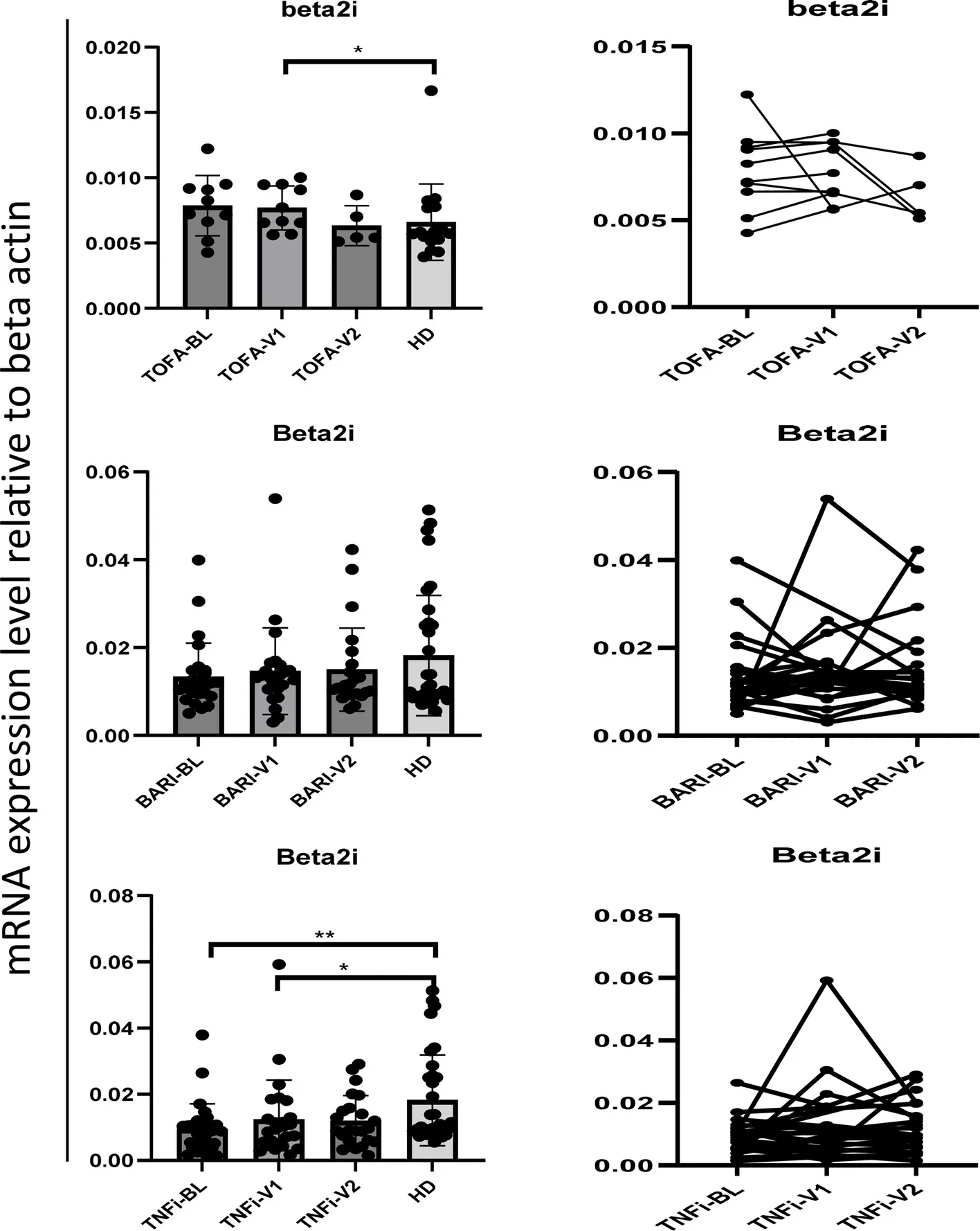
β2i mRNA Expression Under RA Therapies. mRNA expression levels of the proteasome subunit β2i were compared between rheumatoid arthritis (RA) patients treated with tofacitinib, baricitinib, or TNF inhibitors (TNFi)and healthy donors (HD) using the Mann–Whitney U test. Within-patient comparisons of β2i expression were performed using the Wilcoxon matched-pairs signed-rank test: baseline (BL), week 12 (V1), and week 24 (V2) for tofacitinib; week 2 (V1) and week 12 (V2) for baricitinib and TNFi groups. Data are presented as mean ± SD. *P*-values < 0.05 were considered statistically significant. Significance levels are indicated as follows: *p < 0.05, **p < 0.01

Beta2i mRNA was significantly decreased in patients receiving TNFi compared to HD at baseline (*p* = 0.0053); however, at V1 this difference was attenuated (*p* = 0.041), suggesting a relative increase in Beta2i expression over the course of treatment. Similarly, under tofacitinib, beta2i mRNA was significantly elevated in patients compared to HD at V1 (*p* = 0.0355) (Fig. 4, Table 4). Together, these findings suggest that both TNFi and tofacitinib are associated with an upregulation of beta2i mRNA at V1, pointing to a convergent increase in immunoproteasome activity under both treatment modalities.

### SOCS2 protein expression shows partial concordance with transcriptional changes

Given that SOCS2 demonstrated the most consistent treatment-driven changes at the mRNA level across both the tofacitinib and baricitinib cohorts, SOCS2 protein expression was subsequently measured in all three cohorts. At the protein level, SOCS2 expression was significantly reduced at baseline in RA patients treated with Tofacitinib (*p* = 0.0389) and TNFi (*p* = 0.0075) compared with HD. This reduction persisted throughout follow-up in the TNFi group, with SOCS2 protein levels remaining significantly decreased at V1 (*p* = 0.0153) and V2 (*p* = 0.0476).

**Fig. 5:**
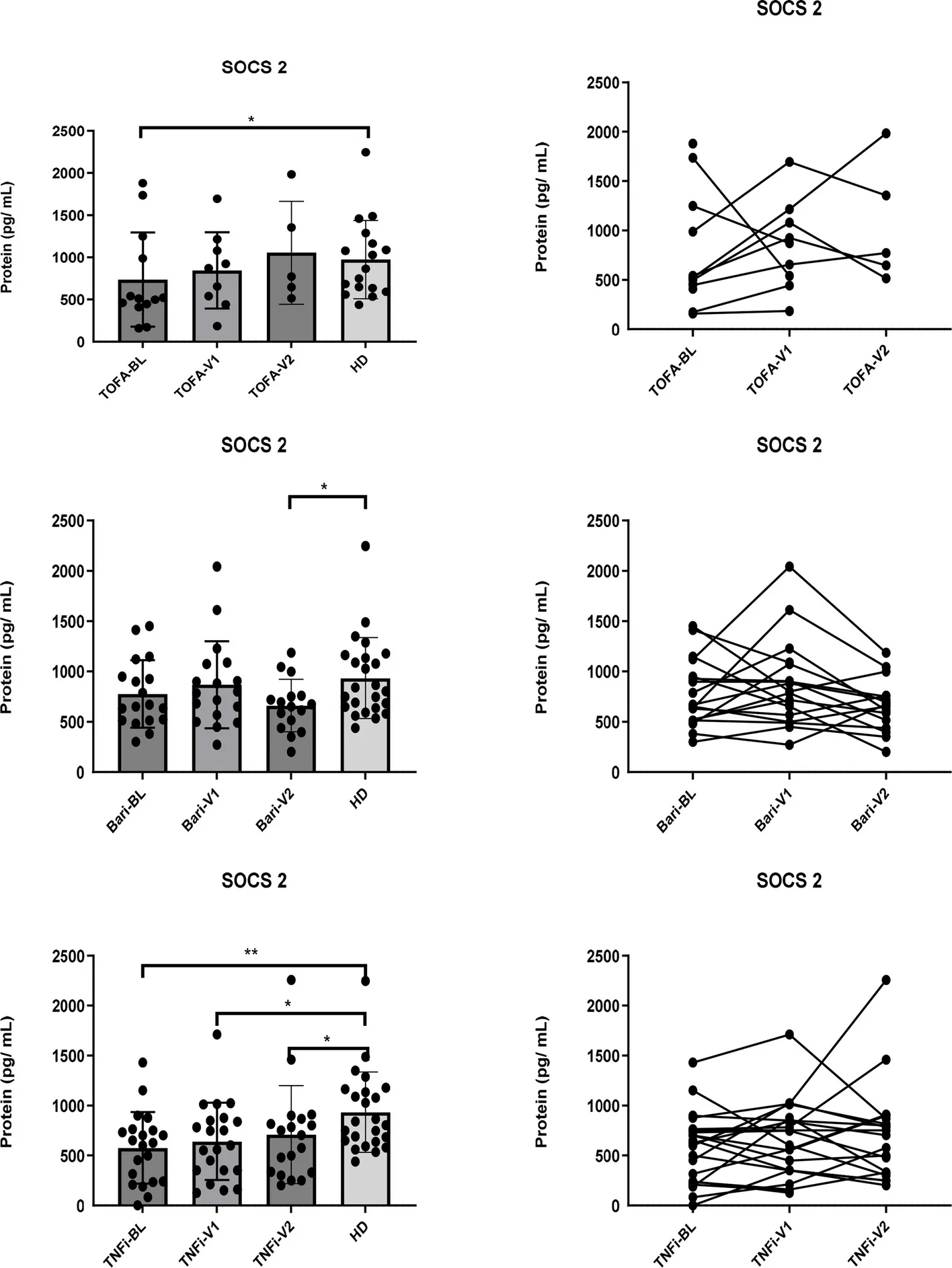
SOCS2 Protein Expression Under RA Therapies. Protein expression levels of SOCS2 were compared between rheumatoid arthritis (RA) patients treated with tofacitinib, baricitinib, or TNF inhibitors (TNFi)and healthy donors (HD) using the Mann–Whitney U test. Within-patient comparisons of SOCS2 expression were performed using the Wilcoxon matched-pairs signed-rank test: baseline (BL), week 12 (V1), and week 24 (V2) for tofacitinib; week 2 (V1) and week 12 (V2) for baricitinib and TNFi groups. Data are presented as mean ± SD. *P*-values < 0.05 were considered statistically significant. Significance levels are indicated as follows: *p < 0.05, **p < 0.01

In contrast, no significant differences compared with HD were observed at follow-up visits in the tofacitinib group, suggesting normalization of SOCS2 protein expression after treatment. Patients treated with Baricitinib exhibited significantly reduced SOCS2 protein levels at V2 compared with HD (*p* = 0.0312). Although no statistically significant longitudinal changes were detected between V1 and V2, a trend toward decreasing SOCS2 protein levels over time was observed (*p* = 0.0595) (Fig. 5, Table 4).

Overall, these findings suggest a time-dependent effect of baricitinib on SOCS2 protein abundance, with significant reduction becoming evident at later time points, while within-group longitudinal changes did not reach statistical significance.

When compared to the mRNA data, SOCS2 showed a consistent treatment-associated decrease at the transcriptional level, particularly under baricitinib and tofacitinib, whereas protein levels remained more stable over time and did not show a clear corresponding longitudinal decline. This partial discordance between mRNA and protein dynamics suggests that SOCS2 regulation may involve post-transcriptional mechanisms, and that changes in mRNA expression are not directly mirrored by proportional changes in protein abundance over the studied time course.

A summary of statistical significance is presented in Table 4.

### Association between gene expression and treatment response

To investigate whether analyzed gene expression was associated with treatment response, patients were classified as responders (R) or non-responders (NR) at V1 and V2 according to the change in DAS28-ESR from baseline. Differences in baseline gene expression and longitudinal changes between responders and non-responders were assessed using the Mann–Whitney U test. To determine whether molecular changes reflected the magnitude of clinical improvement, correlations between changes in gene expression and changes in DAS28-ESR or HAQ were analyzed using Spearman’s correlation analysis.

Among all analyzed genes, SOCS3 was the only marker consistently associated with responder status.

In the baricitinib cohort, baseline SOCS3 gene expression was significantly higher in non-responders than in responders (*p* = 0.0188). In contrast, baseline DAS28-ESR was significantly higher in responders than in non-responders (*p* = 0.0446), indicating that responders entered treatment with greater clinical disease activity despite exhibiting lower baseline SOCS3 expression.

SOCS3 expression decreased significantly during the initial treatment phase in the overall cohort (baseline to V1; *p* = 0.0075), with no further significant changes thereafter. Compared with healthy donors (HD), SOCS3 expression was markedly elevated at baseline (*p* < 0.0001) but was no longer significantly different from HD levels at V1 or V2, suggesting normalization following baricitinib treatment.

**Fig. 6:**
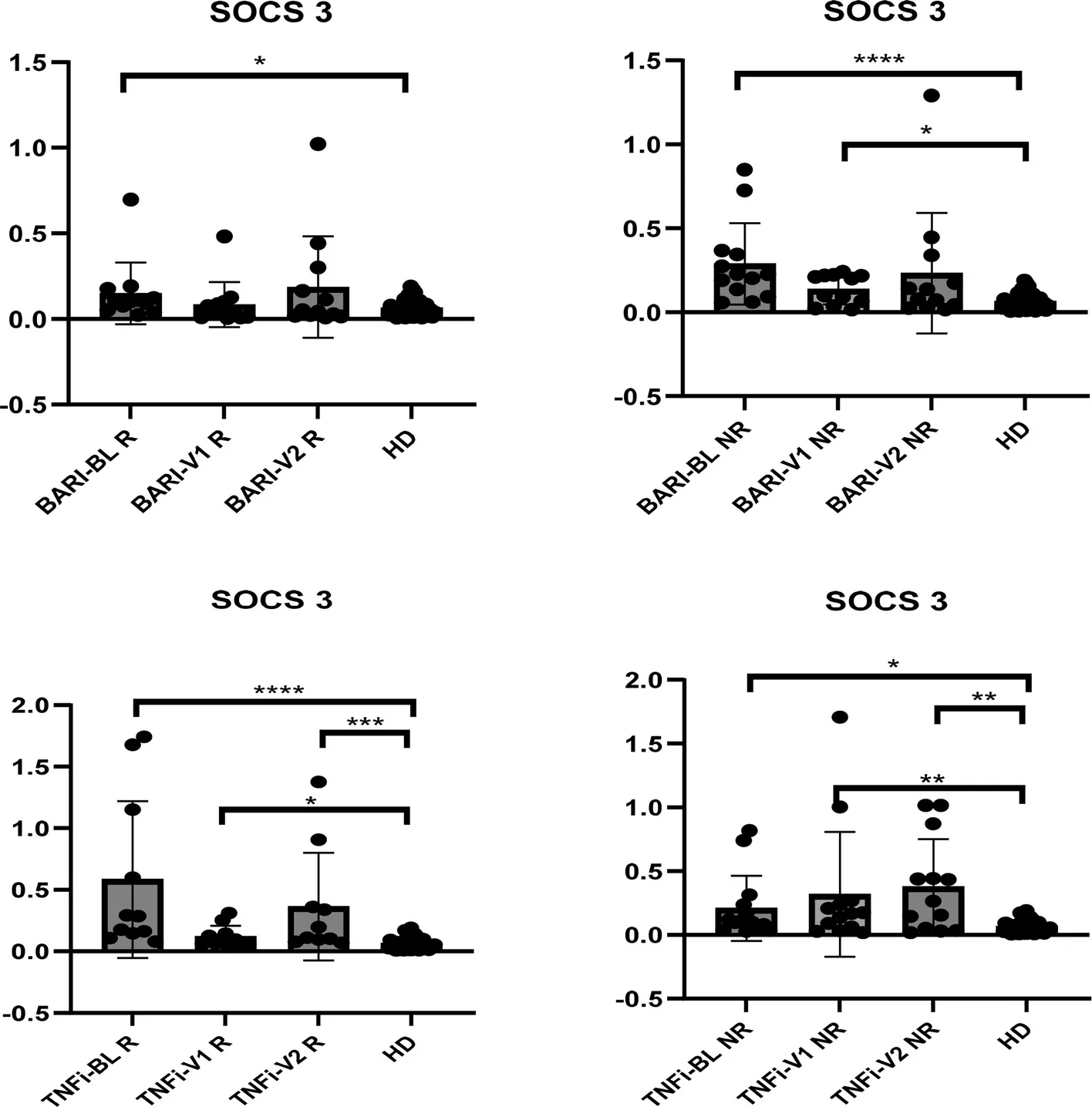
SOCS3 mRNA Expression in Responders and Non-responders During Baricitinib and TNF inhibitors (TNFi) Therapy. SOCS3 mRNA expression was analyzed in the baricitinib and TNFi cohorts, with patients classified as responders (R) or non-responders (NR) according to an improvement in DAS28-ESR of > 1.2 points from baseline. Expression levels were assessed at baseline (BL), visit 1 (V1; 2 weeks), and visit 2 (V2; 12 weeks) and compared with healthy donors (HD). Statistical comparisons between groups were performed using the Mann–Whitney U test. Data are presented as mean ± SD. *P* values < 0.05 were considered statistically significant. Significance levels are indicated as follows: *p < 0.05, **p < 0.01, ***p < 0.001, ****p < 0.0001

When stratified by treatment response, the reduction in SOCS3 expression from baseline to V1 did not differ significantly between responders and non-responders. Among responders, SOCS3 expression was significantly elevated only at baseline (*p* = 0.020 vs. HD), whereas expression at V1 and V2 was comparable to HD levels. In contrast, non-responders showed significantly elevated SOCS3 expression at both baseline (*p* < 0.0001 vs. HD) and V1 (*p* = 0.0128 vs. HD), with normalization observed by V2 (Fig. 6, Table 6).

In the TNFi cohort, baseline SOCS3 expression was significantly higher in responders than in non-responders (*p* = 0.0384), whereas baseline DAS28-ESR did not differ significantly between the two groups. SOCS3 expression remained markedly elevated compared with healthy donors (HD) throughout follow-up (baseline, V1, and V2: all *p* < 0.0001), indicating persistent dysregulation during TNFi therapy.

Although longitudinal analysis of the entire cohort showed no significant reduction in SOCS3 expression at V1 or V2, response-stratified analysis revealed a significantly greater reduction from baseline to V1 in responders than in non-responders (*p* = 0.0059). However, SOCS3 expression in responders remained significantly higher than in HD at all time points (baseline: *p* < 0.0001; V1: *p* = 0.0308; V2: *p* = 0.0003), indicating only partial normalization during TNFi treatment. In contrast, non-responders showed little change or even an increase in SOCS3 expression over the same period, remaining significantly elevated compared with HD at baseline (*p* = 0.0169), V1 (*p* = 0.0067), and V2 (*p* = 0.0029).

Correlation analyses further supported the biological relevance of these findings. In baricitinib responders, changes in SOCS3 expression from baseline to V2 correlated significantly with changes in DAS28-ESR (Spearman’s *r* = 0.6196, *p* = 0.0205), indicating that greater reductions in SOCS3 expression were associated with greater clinical improvement. Significant correlations with ΔDAS28-ESR were also observed for SOCS1 (*r* = 0.7078, *p* = 0.0060) and CIS1 (*r* = 0.5733, *p* = 0.0347), suggesting that modulation of multiple components of the JAK/STAT pathway was associated with therapeutic response. In baricitinib non-responders, changes in SOCS2 expression correlated negatively with ΔHAQ (*r* = − 0.8001, *p* = 0.0397). Similarly, in TNFi responders, changes in SOCS1 expression correlated positively with ΔHAQ (*r* = 0.5283, *p* = 0.0451).

Overall, SOCS3 was the only gene consistently associated with treatment response across both treatment cohorts. Baseline SOCS3 expression distinguished responders from non-responders, while longitudinal SOCS3 dynamics reflected treatment-specific molecular responses. Furthermore, reductions in SOCS3 expression correlated with clinical improvement during baricitinib therapy. Normalization of SOCS3 expression toward HD levels occurred rapidly following baricitinib treatment but remained incomplete during TNFi therapy, highlighting distinct treatment-specific effects on SOCS3 regulation. Collectively, these findings identify SOCS3 as the strongest response-associated biomarker among the genes analyzed. Baseline characteristics according to treatment response and a summary of the statistical analyses for the baricitinib and TNFi cohorts are provided in Tables 5 and 6, respectively.

**Table 5:** Baseline demographic and clinical characteristics of responders and non-responders in the TNF inhibitor (TNFi) and baricitinib cohorts

Characteristic	Baricitinib Responders (*n* = 12)	Baricitinib Non-responders (*n* = 14)	TNFi Responders (*n* = 11)	TNFi Non-responders (*n* = 14)
Female, n (%)	8 (66.7%)	10 (71.4%)	7 (63.6%)	11 (78.6%)
Male, n (%)	4 (33.3%)	4 (28.6%)	4 (36.4%)	3 (21.4%)
Age, female (years)	59.6 ± 8.6	54.9 ± 14.8	58.6 ± 15.7	64.2 ± 7.3
Age, male (years)	62.8 ± 5.0	52.8 ± 8.8	50.0 ± 10.0	62.3 ± 18.0
Prednisolone co-therapy, n (%)	10 (83.3%)	10 (71.4%)	7 (63.6%)	6 (42.9%)
Methotrexate co-therapy, n (%)	4 (33.3%)	3 (21.4%)	4 (36.4%)	3 (21.4%)
Opioid use, n (%)	1 (8.3%)	1 (7.1%)	0 (0%)	0 (0%)
NSAID use, n (%)	9 (75.0%)	8 (57.1%)	6 (54.5%)	5 (35.7%)
Previous biologic therapy, n (%)	9 (75.0%)	11 (78.6%)	2 (18.2%)	2 (14.3%)
Disease duration (years)	10.50 ± 12.07	9.29 ± 7.27	5.45 ± 4.27	8.12 ± 8.29
Baseline DAS28-ESR	5.61 ± 1.42	4.56 ± 1.04	5.96 ± 1.02	5.31 ± 1.16
Baseline HAQ	1.23 ± 0.84	1.19 ± 0.66	1.09 ± 0.80	1.26 ± 0.59

**Table 6:** Summary of statistical analyses of SOCS3 expression according to treatment response in the baricitinib and TNF inhibitor cohorts. Visit 1 (V1) and visit 2 (V2).; — = not significant, HD: Healthy donors

Analysis	Baricitinib	TNF inhibitors
Responders vs. Non-responders (Mann–Whitney U test)		
Baseline SOCS3	*p* = 0.0188 * ↑ in non-responders	*p* = 0.0384 * ↑ in responders
Baseline DAS28	*p* = 0.044 ↑ in responders	—
ΔSOCS3 (Baseline → V1)	—	*p* = 0.0059 ** ↑ in responders
Longitudinal changes within patients (Wilcoxon matched-pairs test)		
Baseline → V1	*p* = 0.0075 ** ↓	—
Baseline → V2	—	—
V1 → V2	—	—
Patients vs. Healthy Donors (Mann–Whitney U test)		
Baseline vs. HD	*p* < 0.0001 **** ↑ in patients	*p* < 0.0001 **** ↑ in patients
V1 vs. HD	—	*p* < 0.0001 **** ↑ in patients
V2 vs. HD	—	*p* < 0.0001 **** ↑ in patients
Responders vs. Healthy Donors		
Baseline (R) vs. HD	*p* = 0.020 * ↑ in responders	*p* < 0.0001 **** ↑ in responders
V1 (R) vs. HD	—	*p* = 0.0308 * ↑ in responders
V2 (R) vs. HD	—	*p* = 0.0003 *** ↑ in responders
Non-responders vs. Healthy Donors		
Baseline (NR) vs. HD	*p* < 0.0001 **** ↑ in non-responders	*p* = 0.0169 * ↑ in non-responders
V1 (NR) vs. HD	*p* = 0.0128 * ↑ in non-responders	*p* = 0.0067 ** ↑ in non-responders
V2 (NR) vs. HD	—	*p* = 0.0029 ** ↑ in non-responders
Correlation with clinical response (Spearman’s correlation)		
ΔSOCS3 (Baseline → V2) vs. ΔDAS28 (responders)	*r* = 0.6196, *p* = 0.0205	—
Overall interpretation		
Normalization toward HD	Rapid normalization by V1 (responders)	Partial normalization only

*p < 0.05, **p < 0.01, ***p < 0.001, ****p < 0.0001. ↑ increase, ↓ decrease

**Fig. 7:**
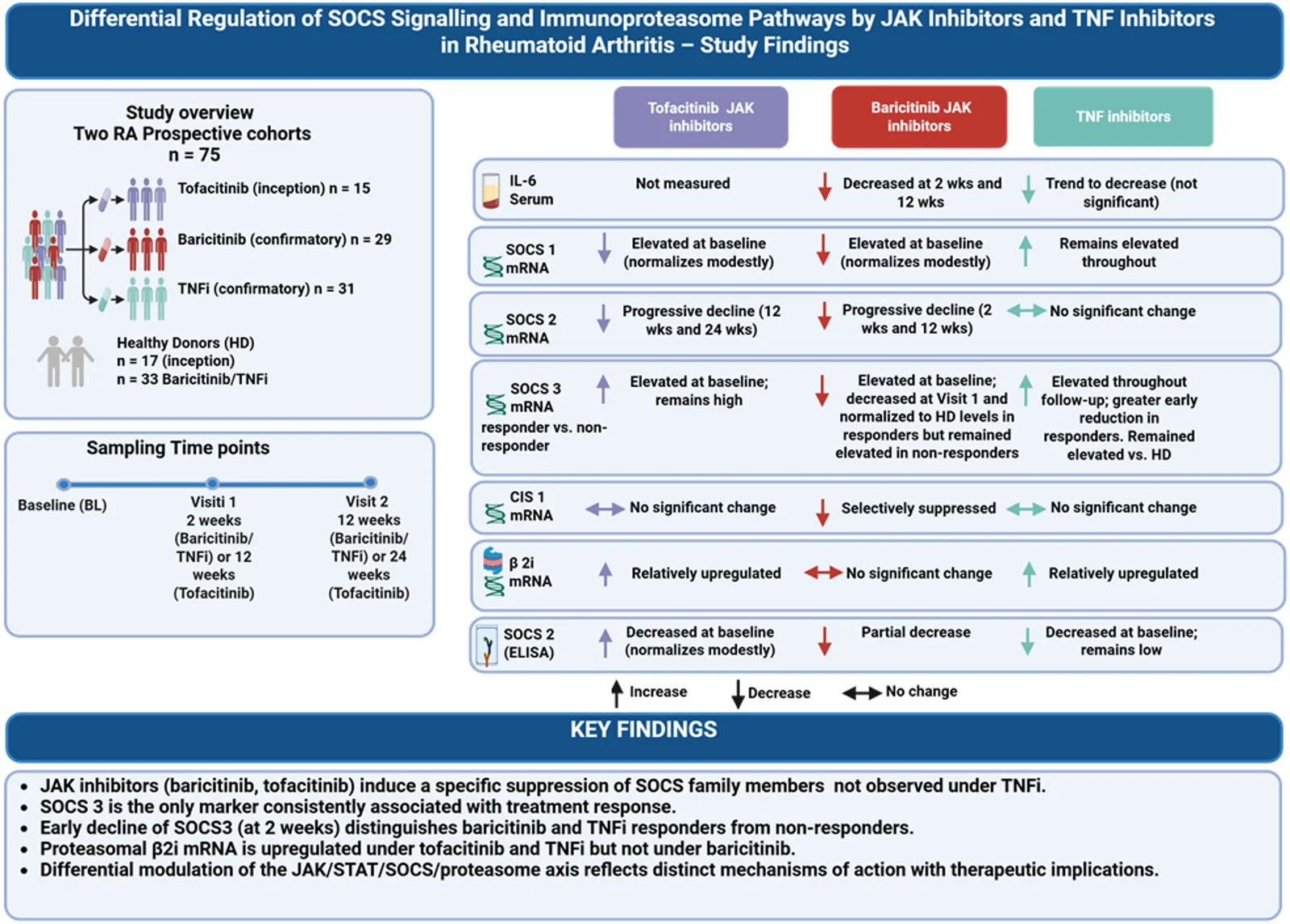
Graphical Summary Illustrating the Differential Effects of Baricitinib, Tofacitinib, and TNF Inhibitors on the SOCS Signaling Pathway, Immunoproteasome β2i, and IL-6

A graphical summary illustrating the differential effects of baricitinib, tofacitinib, and TNFi on the SOCS signalling pathway, immunoproteasome β2i, and IL-6 is presented in Fig. 7.

## Discussion

This pilot study systematically characterises the molecular effects of JAK inhibition and TNFi on the JAK/STAT/SOCS/proteasome axis in RA patients, revealing treatment-specific signatures in SOCS family expression and immunoproteasome regulation that may help explain differential clinical and analgesic responses across these therapeutic classes.

### Clinical response and disease activity

All three treatment groups achieved comparable responder rates at both early (V1) and late (V2) time points, consistent with findings from network meta-analyses demonstrating broadly similar efficacy among approved bDMARDs and JAKi in biologic-naïve RA patients [16]. The progressive and significant reduction in DAS28-ESR observed under baricitinib, accompanied by significant IL-6 suppression at both time points, aligns with its established mechanism of JAK1/2 blockade impairing IL-6 receptor signal transduction [12, 24]. Notably, TNFi produced robust clinical improvements without significantly reducing circulating IL-6, consistent with prior observations that TNF and IL-6 pathways are partially independent and that residual IL-6 signalling may persist under TNF monotherapy [14].

### SOCS family regulation: a JAKi-specific signature

The most consistent molecular findings concern the SOCS family, which showed treatment-specific patterns clearly distinguishing JAK inhibition from TNFi. SOCS2 mRNA exhibited the most robust and progressive suppression under both baricitinib and tofacitinib, with no significant change under TNFi. SOCS proteins are classical negative feedback regulators of JAK/STAT signalling, induced by cytokine stimulation and acting through SOCS-box-mediated proteasomal targeting of activated JAKs [6, 25]. The selective suppression of SOCS2 under JAK inhibitors most likely reflects reduced cytokine-driven JAK/STAT transcriptional activity, since SOCS genes are canonical STAT target genes and their expression is tightly coupled to upstream pathway activation [6]. The absence of this effect under TNFi, which operates upstream of JAK/STAT through TNFR-mediated NF-κB signalling, underscores the pathway specificity of this observation.

SOCS1 and SOCS3 were significantly elevated at baseline in both baricitinib- and TNFi -treated cohorts compared to HD, reflecting chronic immune activation characteristic of active RA [10, 11]. Under baricitinib, both SOCS1 and SOCS3 declined within patients over time, suggesting progressive normalisation of the cytokine feedback landscape with sustained JAK1/2 blockade. In contrast, SOCS3 elevation persisted throughout the observation period in the TNFi group, implying that TNFi does not suppress — and may sustain — SOCS3 upregulation driven by residual cytokines acting through JAK-dependent pathways, such as IL-6 and IL-17. This observation is consistent with the known redundancy of pro-inflammatory cytokine networks in RA synovium and the incomplete suppression of upstream signalling achieved by single-cytokine targeting [3].

CIS1, another JAK/STAT target and competitive inhibitor of cytokine receptor–STAT interactions [6], was selectively reduced under baricitinib, suggesting more complete attenuation of JAK1/JAK2-mediated transcription by this agent compared to either tofacitinib or TNFi. This may reflect the pharmacological selectivity of baricitinib for JAK1/2, which are the principal transducers of signalling through the shared gp130 subunit used by IL-6-family cytokines, as well as through type I/II interferon receptors [24].

### Discordance between SOCS2 mRNA and protein

An intriguing finding is the partial uncoupling between SOCS2 transcriptional and protein dynamics. While mRNA levels declined rapidly and substantially under JAK inhibition, protein levels showed more attenuated and delayed changes, not reaching statistical significance as a longitudinal trend within the baricitinib group. This mRNA–protein discordance is not without precedent in the SOCS literature. SOCS proteins are themselves regulated post-transcriptionally, including through microRNA-mediated translational repression, protein–protein interactions with elongin BC complexes, and proteasomal degradation [7, 26]. It is therefore plausible that under conditions of reduced transcriptional drive, compensatory post-translational mechanisms stabilise residual SOCS2 protein, creating a temporal buffer between gene expression changes and functional protein levels. This possibility merits investigation in future studies examining SOCS2 protein half-life and ubiquitination dynamics under JAK inhibitor treatment.

### Immunoproteasome regulation

The immunoproteasome subunit β2i showed divergent regulation across treatment modalities. Relative upregulation of β2i mRNA was observed under both tofacitinib at Visit 1 and in TNFi -treated patients over time. The immunoproteasome is assembled in response to inflammatory cytokines — particularly IFN-γ and TNF — and serves dual roles in enhancing MHC class I antigen presentation and in mediating SOCS-dependent JAK protein degradation [9, 27]. The convergent increase in β2i under both tofacitinib and TNFi is noteworthy given their distinct mechanisms, and may represent a shared compensatory response to persistent or redistributed cytokine stimulation. The absence of a corresponding β2i increase under baricitinib might reflect more effective suppression of the full JAK-dependent cytokine cascade that drives immunoproteasome assembly, though this interpretation requires confirmation in larger studies.

### SOCS3 as a predictor of treatment response

Among all analysed genes, SOCS3 emerged as the only marker consistently associated with treatment response, highlighting its potential clinical relevance for patient stratification. Baseline SOCS3 expression distinguished responders from non-responders in both the baricitinib and TNFi cohorts, although the direction of this association differed according to treatment modality. In the baricitinib cohort, baseline SOCS3 expression was significantly higher in non-responders than in responders. In contrast, TNFi responders exhibited higher baseline SOCS3 expression than non-responders. These findings suggest that the association between pretreatment SOCS3 expression and subsequent clinical response differs between JAK inhibition and TNF blockade.

Longitudinal analyses further demonstrated distinct treatment-specific SOCS3 dynamics. Baricitinib induced rapid normalization of SOCS3 expression toward healthy donor levels, particularly in responders. Under TNFi therapy, responders showed a significantly greater early decline in SOCS3 expression than non-responders; however, this reduction resulted in only partial normalization despite clinical improvement. SOCS3 is known to exert broad inhibitory effects on multiple cytokine pathways, including IL-6, IL-17, and G-CSF signalling, and its sustained elevation may indicate a state of cytokine feedback dysregulation and persistent inflammatory activation [28, 29]. Importantly, changes in SOCS3 expression during baricitinib treatment correlated significantly with improvements in DAS28-ESR, further supporting the biological relevance of SOCS3 regulation during therapy.

Collectively, our findings identify SOCS3 as the strongest response-associated biomarker among the analysed genes and suggest that both baseline expression and early longitudinal dynamics may provide clinically relevant information on therapeutic response. Nevertheless, these findings should be considered exploratory because of the relatively small subgroup sizes and require validation in larger, independent prospective cohorts before clinical implementation.

### Implications for the analgesic effects of baricitinib

The pronounced analgesic effect of baricitinib, which emerges within days of treatment initiation and exceeds that observed with TNFi [12, 30], is not fully explained by anti-inflammatory mechanisms alone. The present data, showing progressive suppression of SOCS family members specifically under baricitinib, raise the possibility that modulation of neuroinflammatory JAK signalling may contribute. JAK1/JAK2 are expressed in dorsal root ganglia and spinal cord neurons, and cytokine-driven JAK/STAT activation has been implicated in central and peripheral sensitisation in chronic pain states [15, 31]. Whether the SOCS-level changes observed under baricitinib reflect broader neuroinflammatory pathway suppression and thereby contribute to its analgesic properties remains speculative and warrants dedicated investigation in future studies.

### Limitations

Several limitations should be acknowledged. This is a pilot study with a modest sample size, particularly in the tofacitinib cohort, which limits statistical power and precludes definitive conclusions. The two cohorts were recruited and analysed prospectively but sequentially, and not all molecular markers were assessed identically across groups. Furthermore, the confirmatory baricitinib and TNFi cohort focused on a selected panel of genes identified in the inception tofacitinib cohort. Consequently, additional treatment-specific molecular signatures, including other components of the JAK/STAT pathway or cytokine networks, may not have been captured. Furthermore, cytokine analyses were limited to IL-6, which was selected because of its close association with JAK/STAT signalling and its established role in RA pathogenesis. Although IL-6 provides relevant mechanistic information, assessment of a broader cytokine panel would have offered a more comprehensive characterization of treatment-induced immunological changes.

Analyses were performed in PBMCs, which, while accessible and informative, do not directly represent the synovial compartment where pathological inflammation is most active. Furthermore, the responder/non-responder analyses, including the association between SOCS3 expression and treatment response, were exploratory and based on relatively small subgroup sizes. These findings require validation in appropriately powered, ideally randomised cohorts before their predictive value can be established. Finally, treatment response was defined as an improvement in DAS28-ESR of > 1.2 points rather than according to the EULAR response criteria. Although this threshold reflects a clinically meaningful improvement in disease activity, it may limit direct comparison with other studies using standard EULAR classifications. Future studies incorporating larger patient populations, multivariable analyses, comprehensive molecular profiling, and standardized response criteria will be important to further establish the predictive value and clinical utility of SOCS-related biomarkers in rheumatoid arthritis.

## Conclusion

This study identifies a JAK inhibitor-specific molecular signature in RA, characterised by progressive suppression of SOCS2, SOCS1, SOCS3, and CIS1 mRNA — effects not observed under TNFi. The partial discordance between SOCS2 mRNA and protein levels suggests post-transcriptional regulation as an additional layer of pathway control.

Among all analyzed genes, SOCS3 emerged as the only marker consistently associated with treatment response. Pretreatment SOCS3 expression distinguished responders from non-responders, while longitudinal SOCS3 dynamics reflected treatment-specific molecular responses. Moreover, changes in SOCS3 expression correlated with clinical improvement during baricitinib therapy. Rapid normalization of SOCS3 expression toward healthy donor levels occurred under baricitinib but remained incomplete during TNFi therapy, highlighting distinct effects of JAK inhibition and TNFi on cytokine feedback regulation.

Collectively, these findings identify SOCS3 as the strongest response-associated biomarker among the analyzed genes and provide further insight into how JAK inhibitors and TNFi differentially remodel cytokine signalling pathways in RA. Validation in larger, independent prospective cohorts will be required before these findings can be translated into clinical practice and their utility for treatment stratification can be established.

## Data Availability

No datasets were generated or analysed during the current study.
